# Fibrin Glue Coating Limits Scar Tissue Formation around Peripheral Nerves

**DOI:** 10.3390/ijms25073687

**Published:** 2024-03-26

**Authors:** Maximilian Mayrhofer-Schmid, Martin Aman, Adriana C. Panayi, Floris V. Raasveld, Ulrich Kneser, Kyle R. Eberlin, Leila Harhaus, Arne Böcker

**Affiliations:** 1 Department of Hand-, Plastic and Reconstructive Surgery, Burn Center, BG Trauma Center Ludwigshafen, Department of Hand- and Plastic Surgery, University of Heidelberg, 69120 Heidelberg, Germany; 2 Hand and Arm Center, Department of Orthopaedic Surgery, Massachusetts General Hospital, Harvard Medical School, Boston, MA 02215, USA; 3 Department of Plastic, Reconstructive and Hand Surgery, Erasmus Medical Center, Erasmus University, 3015 GD Rotterdam, The Netherlands; 4 Division of Plastic and Reconstructive Surgery, Massachusetts General Hospital, Harvard Medical School, Boston, MA 02215, USA; 5 Department of Hand Surgery, Peripheral Nerve Surgery and Rehabilitation, BG Trauma Center Ludwigshafen, 67071 Ludwigshafen, Germany

**Keywords:** peripheral nerve injury, nerve surgery, microsurgery, nerve regeneration, nerve scarring, nerve adhesions, biomaterials

## Abstract

Scar tissue formation presents a significant barrier to peripheral nerve recovery in clinical practice. While different experimental methods have been described, there is no clinically available gold standard for its prevention. This study aims to determine the potential of fibrin glue (FG) to limit scarring around peripheral nerves. Thirty rats were divided into three groups: glutaraldehyde-induced sciatic nerve injury treated with FG (GA + FG), sciatic nerve injury with no treatment (GA), and no sciatic nerve injury (Sham). Neural regeneration was assessed with weekly measurements of the visual static sciatic index as a parameter for sciatic nerve function across a 12-week period. After 12 weeks, qualitative and quantitative histological analysis of scar tissue formation was performed. Furthermore, histomorphometric analysis and wet muscle weight analysis were performed after the postoperative observation period. The GA + FG group showed a faster functional recovery (6 versus 9 weeks) compared to the GA group. The FG-treated group showed significantly lower perineural scar tissue formation and significantly higher fiber density, myelin thickness, axon thickness, and myelinated fiber thickness than the GA group. A significantly higher wet muscle weight ratio of the tibialis anterior muscle was found in the GA + FG group compared to the GA group. Our results suggest that applying FG to injured nerves is a promising scar tissue prevention strategy associated with improved regeneration both at the microscopic and at the functional level. Our results can serve as a platform for innovation in the field of perineural regeneration with immense clinical potential.

## 1. Introduction

Peripheral nerve injuries remain a significant clinical challenge, with only 3% of patients, following nerve transection, estimated to recover normal sensibility, and 25% to achieve motor function recovery in long-term follow-up [1]. Peripheral nerve lesions of any severity, ranging from local demyelination in continuity to complete transection, can lead to scar formation of various degrees within the peripheral nerve, as well as the surrounding tissue, the latter being described as perineural or paraneural scarring [2]. The development of scar tissue and a pro-fibrotic environment around peripheral nerves can have severe consequences on the function of uninjured nerves as well as on the regeneration of injured nerves. It can result in nerve compression, nerve gliding limitation, and ultimately, functional deficits through damage to axonal structures and the following limitation of signal transduction [3]. Furthermore, increased tension on the nerve was described to limit nerve perfusion and, therefore, to decrease the regenerative capacity of the nerve after injury [4]. Continuous nerve compression and impaired nerve gliding lead to persistent inflammation and further scar development [3]. The combination of mechanical compression and tension with decreased perfusion in a pro-fibrotic environment can severely limit peripheral nerve regeneration [2]. This pathomechanism contributes to a debilitating sequelae in affected patients, including pain, numbness, and loss of function [5].

Yet, despite the high incidence of nerve injury and its effects, the adequate prevention and management of perineural scarring remain elusive. Developing treatment options that optimize outcomes is, therefore, important for the peripheral nerve surgeon [6]. Different materials and concepts have been described that limit or prevent fibrosis and scar formation around peripheral nerves. The proposed strategies include systemic treatments such as the use of pharmaceutic substances like calcium antagonists [7] and topical treatments such as the local application of bioreagents and cells [8,9]. The application of various materials around the nerve has also been proposed [10], including autologous materials, like veins [11,12] or mucosal grafts [13], processed xenografts, exemplarily, porcine submucosa extracellular matrix [14], as well as synthetic materials, such as chitosan [15,16] or collagen [17,18]. These sealant materials have been further refined with other bioactive agents like hyaluronic acid [16,19] or mesenchymal stem cells [15,20]. While first developed to improve outcomes after epineural coaptation or as a biomaterial conduit in critical-size nerve gaps, local sealants were later observed to prevent perineural scarring [11,21]. Solid materials, viscous fluids, and gels are described in the literature as well [22,23,24].

One such sealant material is fibrin glue (FG), a plasma-derived biologic concentrate and known hemostatic agent, which has been utilized in peripheral nerve surgery for a variety of purposes. It has been investigated for different indications, including the replacement of epineural sutures [25], the improvement of the tensile strength of epineural sutures [26], or as a conduit for the bridging of nerve defects where no direct repair is possible after peripheral nerve injury [27]. Its application has been described in both peripheral and central neurosurgery [28] and was demonstrated to prevent peridural fibrosis following laminectomy [29]. The mechanisms of action of FG and its potential to prevent adhesion or scar tissue formation are the subject of ongoing research. In this study, we investigate the potential of perineural FG application for the prevention of scar tissue formation around peripheral nerves and, as a consequence of this, the improvement of peripheral nerve regeneration and functional restitution.

## 2. Results

### 2.1. Functional Outcomes

**Figure 1:**
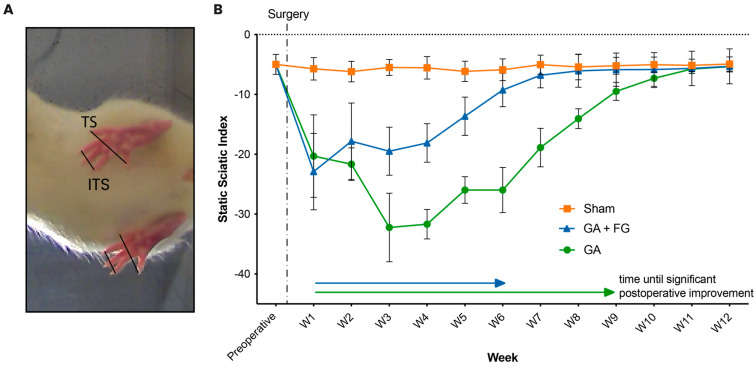
**Functional analysis.** (**A**) The inner toe spread (ITS) and the outer toe spread (TS) were measured and compared between the operated and the not-operated limb. (**B**) The temporal course of the VSSI across the 12-week postoperative period. After a significant decrease in toe spread from the preoperative measurement to the first postoperative measurement for both nerve injury groups (GA and GA + FG), it took the GA + FG group six weeks and the GA group nine weeks to achieve significant postoperative motor function restitution.

The post-surgical functional outcomes were quantified at weekly intervals using the static sciatic index (SSI) throughout the 12-week observation period (Figure 1). In the first week after surgery, a significant decrease in the SSI from the preoperative value (−4.83 ± 1.61) was observed for both the group with glutaraldehyde application only (GA) (−20.3 ± 6.9; *p* = 0.002) and the group with the additional application of FG (GA + FG) (−22.9 ± 6.4; *p* < 0.001). From the first postoperative week on, the SSI in the GA group continued to decrease until it started to improve again after the fourth week. Compared to the first postoperative week, it took the GA group nine weeks to significantly recover (−9.49 ± 1.51; *p* = 0.045). The GA + FG group, on the other hand, started to show an improvement in the SSI from the second postoperative week, with the first significant improvement noted at the six-week time point (−9.26 ± 2.80; *p* = 0.04).

The Sham group showed a significant change in SSI throughout the postoperative observation period. From seven weeks on, the GA + FG group (−6.80 ± 2.10) no longer differed from the Sham group (−5.88 ± 1.81; *p* = 0.24). This equalization effect was seen at 11 weeks for the GA group (GA = −5.75 ± 0.76; Sham = −5.14 ± 1.04; *p* = 0.74).

At the end of the 12-week observation period, neither the GA (−5.35 ± 0.89; *p* = 0.86) nor the GA + FG (−5.31 ± 2.92; *p* = 0.89) group significantly differed from the Sham group (−4.91 ± 1.20).

### 2.2. Histological Scarring Assessment

**Figure 2:**
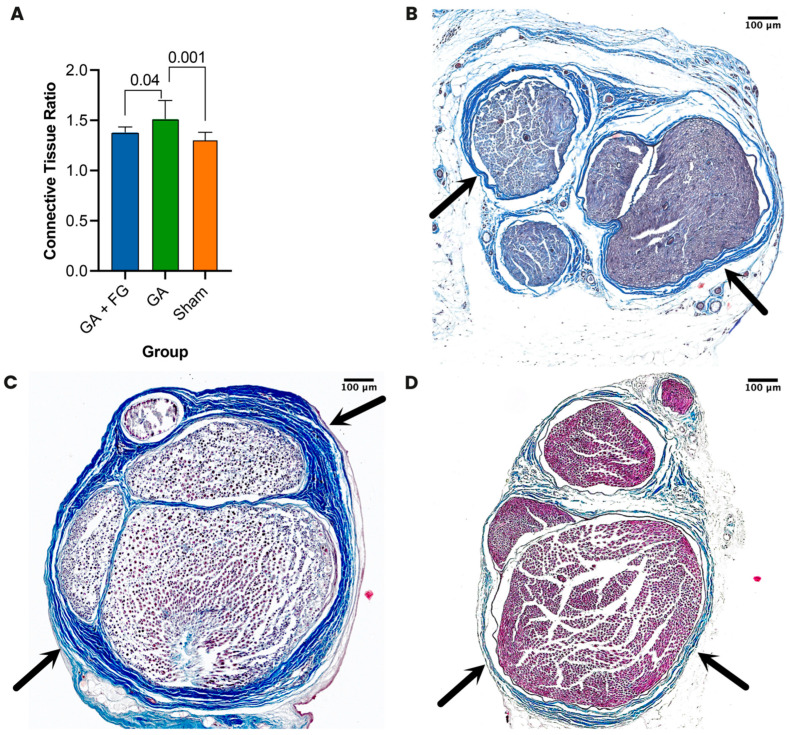
**Histologic assessment of perineural scar tissue formation.** (**A**) Connective tissue ratio. The GA group had the highest ratio, which was significantly different from those of the Sham and the GA + FG groups. (**B**–**D**) Representative cross sections of the sciatic nerve. (**B**) The GA + FG group showed limited scar tissue formation (black arrows) adherent to the nerve after 12 weeks. (**C**) The GA group showed the most extensive epi- and perineural scar tissue formation (black arrows). (**D**) The Sham group showed the lowest scar tissue formation in terms of both epi- and perineurium (black arrows).

Twelve weeks after surgery, the animals were euthanized, their sciatic nerves re-explored, and cross sections of nerve specimens stained using Masson’s trichrome staining. Histologically, we saw higher epi- and perineural scar tissue formation around the sciatic nerve in the GA group compared to the GA + FG group. No observable scarring was seen in the Sham group (Figure 2). The scar tissue was quantified using the connective tissue ratio. We found significantly more scar tissue in the GA group (1.5 ± 0.2) compared to the GA + FG group (1.4 ± 0.1; *p* = 0.045). In the Sham group, we observed no scar tissue formation, with a connective tissue ratio of 1.3 ± 0.09, which was significantly different from that of the GA group (*p* < 0.001) but not from that of the GA + FG group (*p* = 0.23).

### 2.3. Histomorphometric Assessment

**Figure 3:**
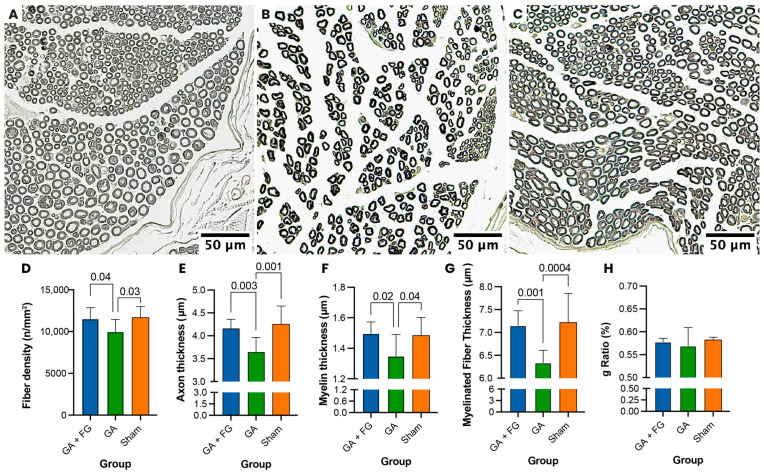
**Histomorphometric analysis.** (**A**–**C**) Representative cross sections of the osmium tetroxide-stained nerves used for histomorphometric analysis. (**A**) The GA + FG group showed dense fibers with thick axons and thick myelin sheaths. (**B**) In the GA group, thinner axons with thinner myelin sheaths were visible. (**C**) The Sham group showed physiological dense fibers with thick axons and thick myelin sheaths. (**D**) Fiber density. The GA group had significantly lower nerve fiber density compared to the other groups. (**E**) Axon thickness. The GA group had significantly lower axon thickness compared to the other groups. (**F**) Myelin thickness. The GA group had significantly lower myelin thickness compared to the other groups. (**G**) Myelinated fiber thickness. The GA + FG and Sham groups had significantly higher myelinated fiber thickness than the GA group. (**H**) The calculated g ratio did not significantly differ between the groups.

Histomorphometric analysis (Figure 3) of osmium tetroxide-stained cross sections from nerve samples taken distally to the injury site revealed significantly higher fiber density in the GA + FG group (11,509 ± 1343 fibers/mm^2^) compared to the GA group (9955 ± 1488 fibers/mm^2^, *p* = 0.047). The fiber density in the Sham group (11,659 ± 1325 fibers/mm^2^) also significantly differed from that of the GA group (*p* = 0.03). The GA + FG group (4.2 ± 0.2 μm) had significantly thicker axons compared to the GA group (3.6 ± 0.3 μm; *p* = 0.003). The results for the Sham group were comparable to those of the GA + FG group (4.3 ± 0.4 μm; *p* = 0.79). The measured myelin thickness was 1.5 ± 0.1 µm for the GA + FG group, which was significantly higher than in the GA group (1.4 ± 0.1 µm; *p* = 0.02). The measured myelin thickness in the Sham group was significantly higher than in the GA group (1.5 ± 0.1 µm; *p* = 0.04).

In terms of myelinated fiber thickness, the GA + FG group (7.1 ± 0.3 µm) had a significantly higher value than the GA group (6.3 ± 0.3 µm; *p* < 0.001). The Sham group (7.2 ± 0.6 µm) also showed a significantly greater myelinated fiber thickness than the GA group (*p* < 0.001). No significant difference was seen between the calculated g ratios of the three groups (0.6 ± 0.0 for all).

### 2.4. Muscle Weight Analysis

**Figure 4:**
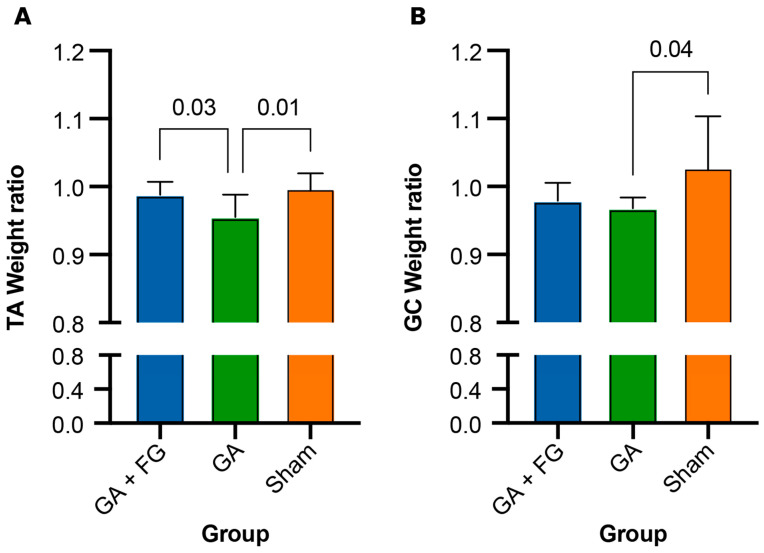
**Muscle weight ratio.** (**A**) The gastrocnemius (GC) muscle of the GA group had the lowest weight ratio. (**B**) Likewise, the tibialis anterior (TA) muscle of the Sham group had the lowest weight ratio.

To assess for potential loss in muscle volume, the wet muscle weight ratio (operated side/non-operated side) was calculated for the tibialis anterior and the gastrocnemius muscles (Figure 4). In terms of the gastrocnemius, we found that the Sham group (1.03 ± 0.08) had a significantly higher wet muscle weight ratio than the GA group (0.97 ± 0.02; *p* = 0.04). No significant differences were detected between the GA + FG (0.98 ± 0.03) group and both the Sham (*p* = 0.12) and the GA groups (*p* = 0.88). Examining the tibialis anterior muscle, a significantly lower muscle weight ratio was seen in the GA group (0.96 ± 0.03) compared to the GA + FG group (0.99 ± 0.02; *p* = 0.03) and the Sham group (0.99 ± 0.03; *p* = 0.01).

## 3. Discussion

The effective prevention of postoperative perineural scar tissue formation has immense clinical potential [5]. The use of biocompatible sealants was previously shown in different preclinical studies to exhibit an inhibitory effect on scar tissue formation [12,13,17,30]. In this study, we demonstrate that FG is one such material that successfully minimizes scar tissue formation around peripheral nerves.

First, in agreement with prior literature, the use of GA was able to induce scar tissue formation around the sciatic nerve of rats [31]. The GA group showed the most compromised nerve histology and histomorphometry, the most impaired nerve function, and the lowest muscle weight. Therefore, the use of GA produces perineural scar formation and leads to impaired axonal parameters and decreased nerve function, further resulting in decreased muscle weight [31].

Overall, this study highlights the potential of FG application. FG was applied around the injured area to test its scar-preventing effects. Twelve weeks postoperatively, the histological analysis of cross sections revealed significantly decreased scar tissue deposition in the animals treated with FG. Histologically stained nerve specimens showed a lower degree of scarring compared to samples from the injured group that did not receive FG, where a thick epineural layer of dense scar tissue was observed. Similarly, Baltu et al. found significantly decreased scar tissue formation on microscopic evaluation after wrapping injured nerves with buccal mucosa in an epineurectomy model [13]. Li et al. described a significantly lower epineurium collagen thickness after the use of chitosan combined with hyaluronic acid after neural crush injury [16]. Kazanci et al. described FG to minimize the development of spinal peridural fibrosis after laminectomy in a preclinical study [29]. Our qualitative and quantitative findings further support the hypothesis of achieving preventive effects on postoperative perineural scarring by the application of sealant materials around peripheral nerves and demonstrate that FG is a viable candidate for this technique.

Furthermore, our results demonstrate improved nerve regeneration in several measured aspects after FG application. In histomorphometric analysis, the FG-treated nerves showed significantly higher fiber density and greater axon thickness, myelin thickness, and myelinated fiber thickness. This effect may be due to an inhibition of scar formation after FG application and, thus, a decrease in the compression of the nerve, in turn preventing ischemia and the degenerative effects exerted by extensive scarring on axonal structures. Following a sciatic nerve crush injury, Li et al. used a chitosan conduit combined with hyaluronic acid to wrap the nerve and described significantly greater axon diameter and myelin sheath thickness compared with the control group [16].

Functionally, immediately after surgery, both groups treated with GA showed significantly lower functional scores, demonstrating the initial nerve injury induced by the application of GA as compared to the Sham group. Furthermore, functional recovery was faster for the GA + FG group compared to the GA group. Both groups had returned to a fully recovered toe spread at the end of the 12-week postoperative observation period. Several mechanisms can be suggested for the improved speed of motor function restitution, including enhanced perfusion as a co-effect of limited scarring [4]. Similarly, Li et al. also found significantly faster functional regeneration in groups treated with a neural conduit, again suggesting not only a scar-preventing mechanism but also a regeneration-supporting effect [16]. Whether improved regeneration and motor function restitution after FG application are solely attributable to the effects of scar tissue formation inhibition or whether the biomaterial properties of FG exert other effects, such as limiting inflammation or promoting perfusion, remains to be investigated.

In terms of the effect on innervated tissues, the wet muscle weight ratio was significantly higher in the tibialis anterior muscle of the GA + FG group compared to the GA group, but a similar effect could not be seen in the gastrocnemius muscle.

A wide variety of sealant materials of synthetic [22,23,32,33], biological [11,13,34], or mixed [15,20] origin have been described, and a small number of these materials are commercially available and have been successfully used in the clinical setting for the management of chronic compressive neuropathies [35]. We hypothesize that the positive effect of this spacer application comes, among other reasons, from preserving the nerve’s ability to glide through the surrounding tissue. Working as a barrier between the nerve and the surrounding muscle and connective tissue, it prevents scarring, leading to direct adhesion, which would decrease nerve mobility [3]. For their future clinical application, spacer materials could be used both for the primary prevention of scar tissue formation after peripheral nerve injuries and as a treatment option for severe or recurrent compression neuropathies. While FG is being used clinically to strengthen and protect coaptation sites [25,26], our findings suggest that a more extensive application on (potential) sites of scarring and compression should be considered to prevent scar tissue formation.

Nerve regeneration can be further influenced by the properties of the material and its effect on the local environment around the nerve. The choice of the material is, therefore, crucial for refining peripheral nerve protection. Specifically, appropriate long-term biocompatibility and biodegradation are essential to avoid processes known to result in increased fibrosis, such as foreign body reactions [36]. The biodegradation of the FG used in this study was previously investigated by Wolbank et al., who described that a subcutaneously implanted FG in a mouse model was no longer detectable 16 days post-implantation using fluorescence imaging and 18 days using histological analysis [37].

Due to its extensively evaluated use in different fields of surgery, including peripheral nerve surgery, FG appears to be suited for use around peripheral nerves. Clinically, its application around peripheral nerves is comparable to the method in this study. Inalöz et al. found its use in nerve coaptation to even induce a lower inflammatory reaction than microsuturing [38]. Furthermore, easy and fast application combined with wide availability support the use of FG for new indications in peripheral nerve surgery.

### Limitations and Further Research

The results of this study need to be considered in light of its limitations. GA-induced nerve injury is a localized form of trauma, and the effect of FG in more severe forms of injury has not yet been studied. Furthermore, the rat sciatic nerve model displays great regenerative capacity, meaning that the results may not be directly translatable to humans, particularly because the regeneration of peripheral nerve defects in humans is a much slower process than in rodents [39,40]. The rat sciatic nerve model is frequently used and highly standardized, but models using larger mammals might offer more comparable insights in the future. Although a highly standardized procedure was applied, variations in regenerative capacity between subjects might have occurred, potentially reflected by the increased standard deviation in the functional outcomes in the first postoperative weeks. In addition, there are limitations to the use of FG itself. This study does not differentiate between the effects caused by the actual material properties of FG and the effects of the sealant technique itself. A comparison of different sealants will be the topic of our future research. Furthermore, as with any liquid materials, FG distribution within the surgical field cannot be precisely controlled. Although special care was taken to avoid this, theoretically, adhesions between the nerve and the surrounding tissue could have formed. In future studies, this could be avoided by applying a separation material between FG and the surrounding tissue until the FG bonding process has been completed. Furthermore, FG and GA could have mixed in their liquid form in situ, thus leading to the incorporation of GA into the solidified FG. While the GA model was initially described for a three-week observation period, a twelve-week evaluation period, used in this study, has the advantage of showing a longer perspective on the effects of FG application. However, the length of the observation period might have had distorted effects which were potentially more present in the initial phase after GA application. It should be noted that, in terms of functional outcomes, an equalization with the Sham group was observed for both injured groups by the 11-week time point. Finally, given the 12-week postoperative evaluation period, this study did not investigate the long-term structural outcomes of FG application.

A transection model could give further insights into the regenerative properties of FG application as a next step for future studies. Hereby, different aspects of functional regeneration including motor, sensory, and autonomic function should be included in the investigation. Additionally, the molecular effects of FG on the development of scar tissue and the regeneration of peripheral nerves should be investigated. Further research should also focus on further refining sealant materials like FG by combining them with bioactive agents with known effects on decreasing perineural scar formation and improving peripheral nerve regeneration using interdisciplinary approaches [41].

## 4. Materials and Methods

### 4.1. Animal Model and Surgical Technique

Ethical approval was obtained from the local ethics board (protocol number: G 20-7-004), and all animal experiments were performed following the Federation of European Laboratory Animal Science Associations (FELASA) guidelines.

**Figure 5:**
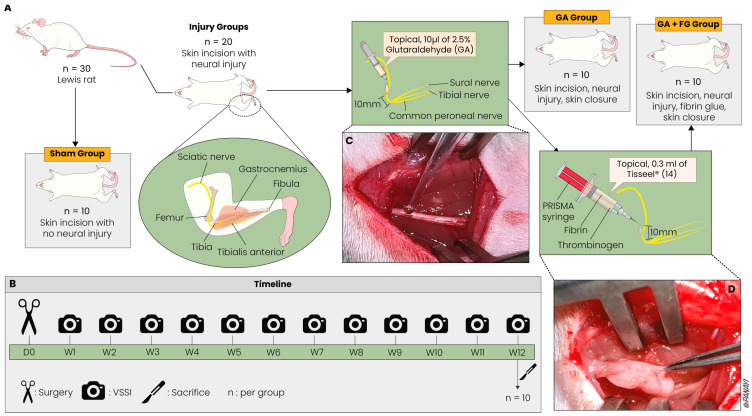
**Experimental setup.** (**A**) Animal groups and rodent anatomy. The injury groups underwent sciatic nerve injury, which was left untreated (GA group) or treated with fibrin glue (GA + FG group). The Sham group underwent exposure of the sciatic nerve without injury. The anatomy of the hind leg and the injury site are illustrated. (**B**) Timeline. The rats were functionally assessed at 12 different time points. At the end of the 12-week period, the rats were sacrificed, and the tissue was prepared for histology. (**C**) In vivo GA application to the exposed sciatic nerve. (**D**) In vivo application of FG and exclusion of adhesion formation with the surrounding muscular tissue.

After an acclimatization period of two weeks, 30 female Lewis rats (weight: 175–200 g) were randomly assigned to three groups of 10 animals each. A previously described sciatic nerve injury protocol was applied to twenty of the rats [31]. Briefly, after exposing the right sciatic nerve with a 15 mm incision along the line between the sciatic notch and the lateral epicondyle of the femur, 10 µL of 2.5% glutaraldehyde (GA) was locally applied onto the sciatic nerve 10 mm proximal to its trifurcation [31]. In 10 of these rats, the sciatic nerve, around the area of GA treatment, was coated equally from all sides along a length of 10 mm with 0.3 mL of FG, five minutes after the application of GA. After the application of FG, a waiting period of three minutes was observed until the bonding process was completed. Care was applied to ensure that FG adhered only to the nerve and not to the surrounding muscular tissue to prevent the iatrogenic impairment of the nerve’s gliding mechanism and allow the nerve to be elevated freely. The other 10 rats received only GA, with no further treatment to the nerve, and underwent skin closure (GA group). Finally, the remaining 10 rats served as the Sham group, whereby the right sciatic nerve was exposed and mobilized, followed by skin closure. All animals were observed across a 12-week postoperative period before undergoing intracardial pentobarbital injection for euthanasia and subsequent specimen extraction. During surgeries and the following analysis, the researchers performing these were blinded to the treatment of each animal. The experimental setup is shown in Figure 5.

### 4.2. Fibrin Glue

The FG used in this experiment was the commercially available, two-component fibrin–thrombinogen tissue adhesive sealant Tisseel^®^ (Baxter Deutschland GmbH, Unterschleissheim, Germany). The sealant was applied locally with a PRISMA syringe (Baxter Deutschland GmbH, Unterschleissheim, Germany), whereby the two components were separately stored in 1 mL containers and were only mixed in the tip of the syringe when applied in situ to prevent premature clotting. The Tisseel^®^ fibrin sealant acts as an intraoperative coagulum and sealant by combining liquid human fibrinogen and artificial aprotinin with human thrombin and calcium chloride, allowing for instant fibrin clothing (“fibrin glue” (FG) formation) [42]. Tisseel^®^ was stored frozen and immediately defrosted in a 37 °C water bath prior to surgery according to the manufacturer’s protocol. A new syringe tip was used for each animal, to ensure consistent conditions for fibrin glue application.

### 4.3. Functional Assessment

A weekly assessment of the sciatic nerve motor function was performed for each animal during the twelve-week postoperative period using the visual static sciatic index (VSSI) [43]. This was done by placing the animals in a plexiglass box with a camera set up at a standardized distance below. Using the camera, pictures were taken when the animals stood still on all four limbs. Using 10 pictures per animal, an average VSSI was calculated by comparing the inner toe spread, that is, the distance between the second and the fourth toe, and the outer toe spread, that is, the distance between the first and the fifth toe of the operated and non-operated hind limb. The measured toe spreads were compared to each other using the formula validated and published by Bozkurt et al. [43].

### 4.4. Histological Assessment

At the 12-week time point, 10 mm long specimens were taken from the injury site of the sciatic nerve, fixated in 4% paraformaldehyde, embedded in paraffin, and sliced into 5 µm thick cross sections. To assess perineural scar formation, samples were stained with Masson’s trichrome according to a standard protocol [44]. Scarring was then quantified by measuring the area of perineural and epineural scar tissue and the area of nerve tissue in cross sections, and the connective tissue ratio was calculated by dividing the area of the nerve and its surrounding scar tissue by the area of only the nerve tissue. Images were taken using the 20× objective of a Zeiss Axio Imager 2 (Carl Zeiss MicroImaging GmbH, Goettingen, Germany).

### 4.5. Histomorphometric Analysis

As scar tissue formation can lead to intraneural damages, assessments for axonal integrity were included in the final analysis. To quantify axonal parameters, a histomorphometric analysis was performed using a standard pre-embedding protocol [45]. Briefly, 3 mm long specimens were taken from the sciatic nerve distal to the injury site. After fixation in 4% paraformaldehyde, the samples were stained with osmium tetroxide and then embedded in paraffin. Subsequently, 2 µm thick cross sections were deparaffinized, and images were taken using the 40× objective of a Zeiss Axio Imager 2 (Carl Zeiss MicroImaging GmbH, Goettingen, Germany).

The cross-sectional area of the whole nerve was measured, and a representative third was chosen for manual analysis. Sampled manual analysis methods have been described in the literature as reliable and, in their results, comparable to complete manual analysis [46]. All axons in this third were counted, and fiber density was calculated after measuring the exact area of this third. Axonal and myelin sheath thickness were measured manually in all fibers in the chosen area. The myelinated fiber thickness, equal to axon thickness plus double myelin thickness, and the g ratio, equal to the inner-to-outer-diameter ratio of a myelinated axon, were calculated from these measurements.

### 4.6. Muscle Weight Assessment

To analyze the effect of sciatic nerve injury on sciatic nerve target muscles, the gastrocnemius muscle and the tibialis anterior muscle were harvested from both the operated and the contralateral side, and the surrounding connective tissue was resected. The muscles were assessed for their wet weight right after excision using an Adventurer Pro analytical scale (Ohaus Europe GmbH, Nänikon, Switzerland). To further quantify the wet muscle weight for a standardized interindividual analysis, the ratio of the operated side weight to the unoperated side weight was calculated and used for comparisons between groups.

### 4.7. Image and Statistical Analysis

For the outcome parameters of the functional, histological, and histomorphometric analyses, images were analyzed, and measurements were made using ImageJ Version 2.3.0/1.53f (RSB, https://imagej.net/ij/, accessed 13 September 2021).

The calculations for the functional, histological, histomorphometric, and wet muscle weight analyses were performed in Microsoft Excel (Microsoft Corporation, Redmond, WA, USA). Normal distribution was assessed using the Shapiro–Wilk test and verified in all datasets. To assess the significance of unmatched grouped datasets, we performed a one-way ANOVA, and a subsequent Tukey’s multiple comparison test was used to compute the *p* values for group-wise comparisons. For the visual static sciatic index (VSSI), a repeated measurements ANOVA with Bonferroni-post hoc test was used to compare measurements at different time points with each other within one group.

Statistical testing was conducted using PRISM GraphPad Version 10.1.1 (GraphPad Software, San Diego, CA, USA) with the threshold for statistical significance set at *p* < 0.05. The data are displayed as mean ± standard deviation. Illustrations were created using Adobe Illustrator 2022 Version 28.3 (Adobe Inc., San Jose, CA, USA).

## 5. Conclusions

Scar tissue formation after peripheral nerve injury is a highly relevant challenge in clinical practice. This study demonstrated that the perineural application of FG following peripheral neural injury significantly reduced perineural scar tissue formation and improved regeneration both at the histological and at the functional level. With its established role in peripheral nerve surgery and its known advantages including ease of application and availability, FG is a promising material to improve peripheral nerve regeneration through the prevention of scar tissue formation clinically. The positive results of FG application shown by this study should serve as a foundation for further research into this material to optimize outcomes following neural injury.

## Data Availability

The data presented in this study are available on request from the corresponding author.

## Abbreviations

FG	Fibrin glue
GA	Glutaraldehyde
GC	Gastrocnemius muscle
SSI	Static sciatic index
TA	Tibialis anterior muscle
VSSI	Visual static sciatic index
